# Contextualising the 2019 E-Cigarette Health Scare: Insights from Twitter

**DOI:** 10.3390/ijerph17072236

**Published:** 2020-03-26

**Authors:** Wasim Ahmed, Xavier Marin-Gomez, Josep Vidal-Alaball

**Affiliations:** 1 Newcastle University Business School, Newcastle University, 5 Barrack Rd, Newcastle upon Tyne NE1 4SE, UK; 2 Health Promotion in Rural Areas Research Group, Gerència Territorial de la Catalunya Central, Institut Català de la Salut, 08272 Sant Fruitós de Bages, Spain; xmarin.cc.ics@gencat.cat (X.M.-G.); jvidal.cc.ics@gencat.cat (J.V.-A.); 3 Servei d’Atenció Primària d’Osona, Gerència Territorial de la Catalunya Central, Institut Català de la Salut, 08500 Vic, Spain; 4 Unitat de Suport a la Recerca de la Catalunya Central, Fundació Institut Universitari per a la recerca a l’Atenció Primària de Salut Jordi Gol i Gurina, 08272 Sant Fruitós de Bages, Spain

**Keywords:** electronic nicotine delivery systems, social media, smoking, twitter

## Abstract

A health scare can be described as a campaign that attempts to alert the public of a particular substance or activity that can lead to a negative effect on health. A recent health scare to emerge relates to the health hazards associated with the use of e-cigarettes, which has caused widespread debate, which peaked towards the end of 2019. Health scares need to be studied in the context in which they occur, and one method of studying them is through social media. This paper identifies two key topics of discussion on Twitter, which consisted of pro-vaping and anti-vaping views. The paper then identifies influential users, frequently occurring words, hashtags, and websites related to this time period in order to gain insight into e-cigarette perceptions. The paper then reviews current scientific evidence and develops a flowchart for the general public, which can be used to for public reassurance and guidance.

## 1. Introduction

Electronic cigarettes or e-cigarettes are handheld battery operated devices that heat liquid and deliver an aerosol that simulates smoking. They may also be referred to as electronic nicotine delivery systems (ENDS) in certain literature. In conjunction with the increased use of these devices [1], a health concern has emerged linking the use of e-cigarettes with pulmonary illness [2,3,4]. The Centers for Disease Control and Prevention (CDC) and the U.S. Food and Drug Administration (FDA) are investigating a multistate outbreak of lung injury associated with the use of e-cigarette or vaping products [5,6]. All of this information is generating widespread debate, which we can be framed as a health scare [7,8] similar to other scares such as the current COVID-19 scare, as well as the H1N1 virus (swine flu), trans fats, Ebola, and the mobile phone usage health scare [9,10]. Health scares can be described as campaigns that attempt to alert the public of a particular substance or activity that can lead to a negative effect on health [11].

Tobacco can be traced back thousands of years and was grown as a crop from around 5000–3000 BC, led by the communities in the Andes, South America (Ram, Nathan, Balraj, 2017). Native Americans would cultivate and smoke tobacco for medicinal and ceremonial purposes. Tobacco eventually spread around other continents and eventually all around the world [12]. Henceforth, it had many thousands of years to become ingrained within human culture and society. In the early years of the 20th Century, cigarette and nicotine marketing through print, television, and radio only helped to boost sales. In recent years, and after the dangers of traditional forms of smoking, e-cigarettes have risen in popularity.

The tobacco industry is argued to be one of the most profitable and deadly industries that exists with cigarette retail values in 2013 hitting $ 722 billion and there being 5.7 trillion cigarettes in circulation [12]. In today’s digital world, it is possible for consumers and the general public to offer their views and opinions across social media platforms. One of the most open platforms to converse about public health is Twitter, where it is also possible to extract tweets for academic research purposes. Previous research has utilised Twitter to identify public views, key discussions, content, and stakeholders related to health scares [13,14,15]. Twitter can be used to study and understand the context of the current e-cigarette health scare. Twitter is an important platform to study because it has the potential to shape mainstream news because tweets can be embedded in online news stories, as well as highlighted in traditional media (such as TV or radio), amplifying their reach. Health scares need to be studied in the context in which they occur, and one method of studying them is through the use of Twitter [10,16]. This is because Twitter provides citizens with a platform that permits the rapid sharing of public views and opinions and allows these views to become viral and highly shared, regardless of their factual truth; henceforth, this is an important and significant area to study. Moreover, the debate around the safety of e-cigarettes is likely to be of interest to a wide variety of stakeholders.

Our study sought to examine an influential time-point related to e-cigarettes from 2019 when there was a heightened interest in e-cigarette safety. We identified a gap in knowledge as no previous empirical work has conducted an analysis of Twitter data related to this time point. Moreover, this is an important topic to study because it aims to build an understanding of how social media may play a role in the global dissemination of amateur and unfounded speculation against accepted medical research. This type of research is increasingly important, as we find medical studies being socially challenged by various social media networks at an increasing rate. We utilised a mix of social network, automated text, and link analysis in order to identify network structures, influential users, the most utilised words, and hashtags. 

We sought to address the following research questions:

1. What was the overall shape of the network structure on Twitter related to the e-cigarette debate?
2. What key themes and/or topics emerged when Twitter users conversing about e-cigarettes on Twitter?
3. Who were the key stakeholders, and what types of content were they sharing on Twitter related to the e-cigarette debate?

A further objective of the study was to review existing literature in order to develop a flowchart for consumers to assess the safety of e-cigarettes by analysing public views from Twitter and by drawing up current advice from health authorities and domain experts. This flowchart is likely to be of interest to consumers and public health agencies across the world.

## 2. Methods 

We retrieved data from Twitter using NodeXL related to the time when the first study was published linking cancer and vaping in mice. NodeXL utilises the Search Application Programming Interface (AP)I. We retrieved data using the keywords “ecigarette” OR “e-cig” OR “ecig” OR “vaping”, and we were able to retrieve a sufficient amount of tweets. The tweets in the network were tweeted over the 17 h, 37 min period from Monday, 07 October 2019, at 21:20 Coordinated Universal Time (UTC), to Tuesday, 08 October 2019, at 14:58 UTC. The graph represents a network of 14,912 Twitter users.

We utilised social network analysis, which is an established method of studying social media content, and a complete overview of network shapes and structures can be found elsewhere [17]. Within our network graph, there was an edge for each “replies-to” relationship in a tweet, an edge for each “mentions” relationship in a tweet, and a self-loop edge for each tweet that was not a “replies-to” or “mentions”. The graph was directed, and vertices were grouped by cluster using the Clauset–Newman–Moore cluster algorithm. The graph was laid out using the Harel–Koren fast multiscale layout algorithm.

## 3. Results

### 3.1. Social Network Analysis of Study Linking Cancer and Vaping Published Research (October 2019)

**Figure 1:**
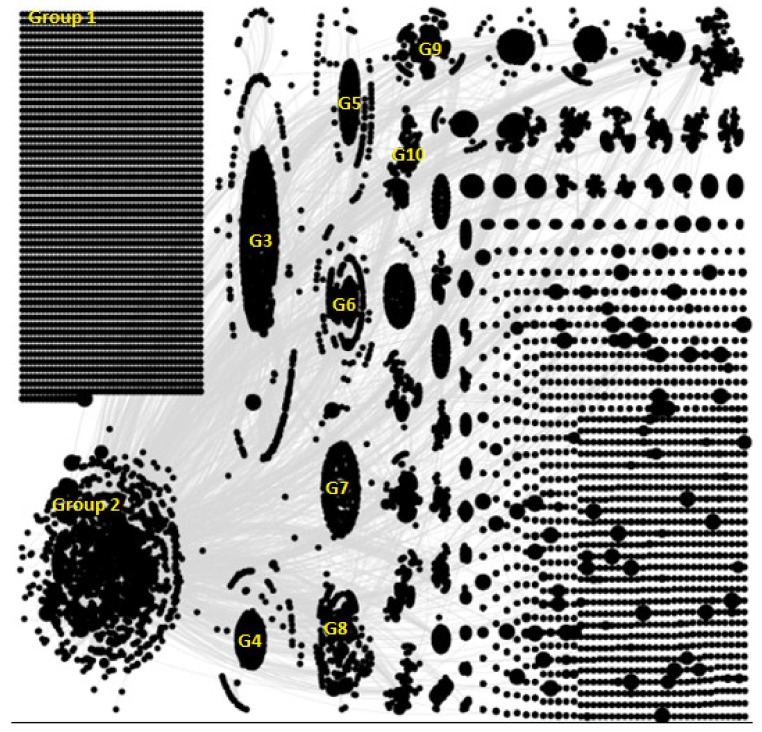
Tweets related to e-cigarettes in October 2019.

**Figure 2:**
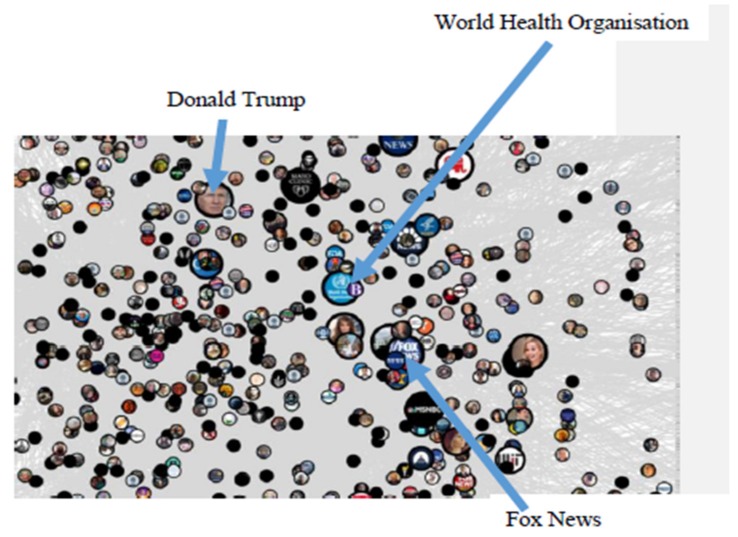
Zooming into Group 2.

Figure 1 below shows a social network analysis of tweets from early October 2019, which relates to a time period when the first academic study was published linking vaping to cancer in mice. Figure 2 is zoomed into Group 2 and labels the influential users within the group. From group 3 onwards we have abbreviated the word ‘Group’ to ‘G’.

The network graph highlights that there were different clusters of discussion taking place on Twitter during this time with two large groups and several smaller groups indicating a number of communities that had emerged related to this topic. The ten largest groups were labelled. There was also a sizeable isolates group (Group 1), which indicated that a number of Twitter users were tweeting about e-cigarettes without mentioning or replying to other Twitter users. In Figure 2, we zoom into Group 2 in which discussions were formed around a number of influential user accounts such as President Donald Trump, the World Health Organisation, and Fox News. Alongside this network graph, we were also able to identify the most frequently used words, hashtags, websites, and most influential users in the network, which are described below.

### 3.2. Most Frequently Occurring Words

Table 1 below displays the most frequently occurring words during this time period. It highlights that mentions of e-cigarettes were made in conjunction with the scientific work that linked them to cancer. 

The most frequently occurring words in Group 1 are displayed in Table 2 below. Here, it can also be seen that many of these words centred on the news story linking vaping to cancer.

The most frequently occurring words in Group 2 (the second largest cluster of Twitter users) are highlighted in Table 3 below. It is important to note that the word-count numbers listed above would also contain hashtags that used those words. 

These words in Group 2 highlighted the polarisation on Twitter as certain hashtags were against the idea of any restrictions on vaping by governments, and Twitter users expressed this through the use of the hashtag “#wevapewevote”, whereas those against e-cigarettes would use hashtags such as “#vapeban”.

### 3.3. Most Frequently Occurring Hashtags

The most frequently occurring hashtags overall are summarized in Table 4 below. These hashtags highlighted division and polarisation on Twitter as some hashtags related to campaigns against vaping such as “vapeban”, whereas other hashtags such as “wevapewevote” were pro-vaping. There were more Twitter users using pro-vaping based hashtags than against them.

### 3.4. Most Frequently Occurring Websites

The top URL shared on Twitter during this time was entitled “Lung Damage From Vaping Resembles Chemical Burns, Report Says” published in the New York Times (*n* = 341) [18]. The other URLs consisted of an article by the Consumer News and Business Channel (CNBC) (*n* = 229) titled Researchers find e-cigarettes cause lung cancer in mice in first study tying vaping to cancer. Further websites also appeared such as an article titled Expert reaction to study on ecig vapour and cancer in mice, which was published by the Science Media Centre (*n* = 124). Interestingly, the article contained reactions from two professors with expertise in tobacco research, and both noted that the study linking e-cigarettes and cancer to mice was potentially seriously flawed in its relevance for human vapers. Another article titled Juul Is Sued by School Districts That Say Vaping Is a Dangerous Drain on Their Resources published by the New York Times was also shared (*n* = 88). These results are summarized in Table 5 below. 

### 3.5. Influential Users Ranked by Betweenness Centrality 

Influential users were ranked by the betweenness centrality algorithm using NodeXL. Influential users consisted of CNBC, which had 3.3 million followers, and the news anchor of Columbia Broadcasting System’s (CBS) Nightly Business Report, who had 31.3 thousand followers. The reason for the prominence of CNBC was because they had published an article on October 7th titled ‘*Researchers find e-cigarettes cause lung cancer in mice in first study tying vaping to cancer’.* A citizen who tweeted the study and noted the findings of the study for the reason for “not following trends” received over 400 retweets and 1.8 thousand likes also became influential and had 6.1 thousand followers. The user was characterizing e-cigarettes as a societal trend with dissent towards those who were using e-cigarettes. A user replied to this tweet indicating that the fear around e-cigarettes seemed irrational when traditional e-cigarettes were still being sold. Other influential accounts consisted of The Centres for Disease Control (CDC) with 1.24 million followers who tweeted about cases of lung damage of users of mostly illicit e-cigarettes containing the Tetrahydrocannabinol (THC), and Gregory Conley, with 18.1 thousand followers, who is a tobacco harm-reduction advocate and supporter of e-cigarettes who was tweeting at this time. Table 6 below provides a summary of the influential users in the network. 

### 3.6. Pro-Vaping and Anti-Vaping Themes

Much of the content around this time related to Twitter users sharing the news story linking vaping and cancer in mice. Furthermore, our tweet clustering, word, and hashtag analysis revealed two further main themes taking place within the overall network related to Twitter users who were, broadly speaking, either pro-vaping or anti-vaping. In order to confirm this finding, we utilised content analysis to categorize tweets until thematic saturation occurred, and we found two additional main themes related to pro-vaping or anti-vaping views. 

Twitter users who were supporting vaping tweeted how using vaping products had helped them stop smoking and also highlighted how the current issue around vaping was very specific to certain illicit types of vaping products that contained Tetrahydrocannabinol (THC). Arguments were also made towards vaping serving a function in harm-reduction as it was comparably safer than smoking. Twitter users against vaping requested for people to stop using e-cigarettes and for tougher legalisation on vaping products. Below, we provide anonymized tweets related to these two main themes.

### 3.7. Pro-Vaping Tweet Extracts 

There were a number of Twitter users who would share their own positive personal experiences of using e-cigarette devices:

*“I was a heavy smoker for many decades and knew I’d be dead in my 50 s and never get a chance to see my grandchildren. But then 5 years ago I started to use e-cigarettes and I may be able to see my grandchildren now”*

Other Twitter users were concerned about the increased focus on younger people using e-cigarettes:

*“The dangers of vaping and youth is completely overblown! Car crashes are actually the leading cause of death in younger people”*

There were also Twitter users who noted the dangers of cigarettes compared to e-cigarettes:

*“Right then, they will ban e-cigs because it causes deaths, but regular cigarettes don’t seem to be a problem, lol!”*

There were also Twitter users who criticised the study linking cancer and vaping:

*“If they re-ran the study and compared vaping and real cigarettes then vaping would be the safer alternative! #vapingsavedmylife”*

In the above tweet extract, the user employed the hashtag “vapingsavedmylife”, which was used by Twitter users to indicate the life-saving potential of vaping.

### 3.8. Anti-Vaping Tweet Extracts 

There were also Twitter users who called for people to quit vaping:

*“Please stop vaping! – read this news story Lung Damage From Vaping Resembles Chemical Burns, Report Says”*

Other Twitter users called for parents to inform their children that e-cigarettes were unsafe:

*“Let your children know that vaping is NOT SAFE, doesn’t matter what their friends say, they’ve been brainwashed by big Tobacco!”*

There were also a number of general comments noting that e-cigarette use was likely to be dangerous:

*“I am not a medical professional, but most likely that vaping is not good for you”*

Another Twitter user noted:

*“I used to smoke 50 cigarettes a day and managed to just quit, that’s the best way to quit. Vaping is not safe.”*

From the tweet extracts above, we can see that there was a diverse range of views and comments noting that e-cigarettes were unsafe. 

## 4. Discussion

**Figure 3:**
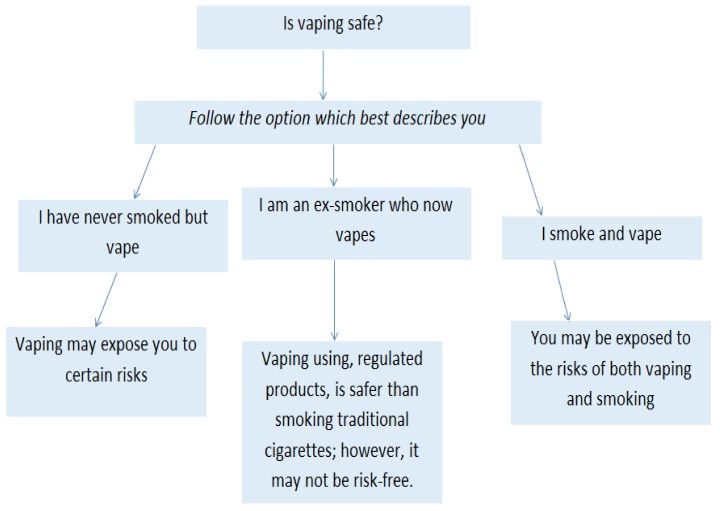
Flowchart based on current by Centers for Disease Control, the NHS, and Cancer Research UK [20,21,22].

This study showed the effects of a research study linking cancer in mice to vaping on Twitter. The study was tweeted by several mainstream media outlets and dominated discussions of e-cigarettes on Twitter. The study, however, was questioned by several experts as they noted that the study conducted on mice could not be generalised to humans. Our study highlighted the impact of academic research on Twitter when it is disseminated through the mainstream media. The publication of the study caused great concern over the safety of e-cigarettes on Twitter, and users of the platform were quick to point out that e-cigarettes were still a much safer alternative to traditional smoking. For instance, health authorities such as the National Health Service in the United Kingdom still noted that e-cigarettes contained a fraction of the risk when compared to that of traditional cigarettes [19]. However, the publication of the study linking cancer to mice may have incorrectly been interpreted and understood by some members of the public who would now believe that e-cigarettes were just as unsafe and/or more unsafe when compared to traditional tobacco based cigarettes. Current advice provided by Centers for Disease Control [20,21,22] notes that e-cigarettes are likely to be safer than smoking traditional cigarettes. Our recommendation to health authorities is to offer guidance to the public in order for citizens to make informed decisions about whether e-cigarettes are safe to consume. A key aspect of assessing whether vaping is safe depends on whether an individual is already engaged in using traditional cigarettes and/or rolled tobacco products. However, information disseminated to consumers around the safety of e-cigarettes can be confusing and vague. Moreover, we found that Twitter users tended to be unsure about the safety of e-cigarettes. Henceforth, based on a review of the literature and the advice provided by the Centers for Disease Control, the National Health Service (NHS), and Cancer Research UK [20,21,22], we developed a flowchart, as shown in Figure 3, that could be utilised by consumers and medical professionals when making a decision about whether e-cigarettes are likely to be safe or unsafe based on their circumstances. 

By analysing public comments on Twitter, we were able to identify potential points of confusion by consumers, and combining our results with that of current medical advice led to the development of the flowchart above. Social media may be a source of information for a subset of the population, and it is important to analyse this information from a public health surveillance perspective. Our findings can also be contrasted with previous empirical work in this area. A study published in 2018 [23] conducted an analysis of e-cigarette discussions on Twitter from 2012 to 2015, which examined the reasons for using such devices. The study found that one of the most popular reasons for using e-cigarettes was to quit traditional cigarettes, and Twitter users were positive towards e-cigarette devices. Moreover, they found that 43% of Twitter users were able to give up traditional cigarettes, which demonstrated the potential of e-cigarettes for smoking cessation. The findings of this study were similar to our present study, which also found that there was a sizeable “pro-vaping” community on Twitter. A further study [24] published in 2014 looked at a specific case study containing tweets for and against public health policy related to the Chicago Department of Public Health. This study also found pro- and anti-e-cigarette views such as that they promote smoking cessation, but also that they are harmful and foster nicotine addiction. A further study in the United Kingdom [25] surveyed 3538 current and 579 recent smokers in November and December 2012. It was also found that there were mixed views among the public about whether e-cigarettes were more or less harmful than traditional cigarettes, with a number of citizens unsure of their safety at the time. These results further highlight the need for easy-to-understand guidance for consumers, and our flowchart developed above will be of interest to health authorities.

## 5. Conclusions

This paper conducted an analysis of current debates surrounding e-cigarette usage on Twitter. We identified two main themes that arose, which were either based on pro-vaping or anti-vaping views, and this method has been utilised in previous research [26]. Our work adds to the body of literature demonstrating the potential of Twitter for health research [27]. We then developed a flowchart, by reviewing existing literature, which can be used to inform public health information dissemination. A limitation of our study is that we examined only English-language tweets, which may have led to more tweets from English-speaking countries such as the United States and the United Kingdom. Furthermore, there may have been some tweets not related to e-cigarettes in the dataset. 

## Figures and Tables

**Table 1:** Most frequently occurring words.

Words	No.
**vaping**	15,452
**cancer**	5181
**cigarettes**	4034
**mice**	3377

**Table 2:** Most frequently occurring words in Group 1.

Words	No.
**vaping**	2177
**cigarettes**	373
**lung**	315
**cancer**	310

**Table 3:** Most frequently occurring words in Group 2.

Words	No.
**vaping**	3452
**# vaping**	1163
**# wevapewevote**	1054
**# vapeban**	807

**Table 4:** Most frequently occurring hashtags.

Words	No.
**vaping**	1459
**wevapewevote**	515
**vape**	424
**vapeban**	391

**Table 5:** Most frequently occurring hashtags.

Title	Publisher	No.
**Lung Damage From Vaping Resembles Chemical Burns, Report Says**	New York Times	341
Researchers find e-cigarettes cause lung cancer in mice in first study tying vaping to cancer	CNBC	229
**Expert reaction to study on ecig vapour and cancer in mice**	Science Media Centre	124
Juul Is Sued by School Districts That Say Vaping Is a Dangerous Drain on Their Resources published	New York Times	88

**Table 6:** Influential users ranked by betweenness centrality.

Rank	Influential User Account	Followers
**1**	CNBC	3.3 million followers
**2**	Anchor of CBS’s Nightly Business Report	31.3 thousand followers
**3**	The Centres for Disease Control	1.24 million
**4**	Citizen	6.1 thousand followers
**5**	Gregory Conley	18.1 thousand followers
